# Theranostic nanoparticles enhance the response of glioblastomas to radiation

**DOI:** 10.7150/ntno.35342

**Published:** 2019-09-17

**Authors:** Wei Wu, Jessica L. Klockow, Suchismita Mohanty, Kimberly S. Ku, Maryam Aghighi, Stavros Melemenidis, Zixin Chen, Kai Li, Goreti Ribeiro Morais, Ning Zhao, Jürgen Schlegel, Edward E. Graves, Jianghong Rao, Paul M. Loadman, Robert A. Falconer, Sudip Mukherjee, Frederick T. Chin, Heike E. Daldrup-Link

**Affiliations:** 1 Department of Radiology, Molecular Imaging Program at Stanford, Stanford University, Stanford, CA, USA; 2 Department of Radiation Oncology, Stanford University, Stanford, CA, USA; 3 Institute of Cancer Therapeutics, Faculty of Life Sciences, University of Bradford, Bradford, UK; 4 Department of Neuropathology, School of Medicine, Technical University of Munich, Munich, Germany

**Keywords:** glioblastoma, glioblastoma initiating cells, theranostic nanoparticle, radiation therapy, imaging, ferumoxytol

## Abstract

Despite considerable progress with our understanding of glioblastoma multiforme (GBM) and the precise delivery of radiotherapy, the prognosis for GBM patients is still unfavorable with tumor recurrence due to radioresistance being a major concern. We recently developed a cross-linked iron oxide nanoparticle conjugated to azademethylcolchicine (CLIO-ICT) to target and eradicate a subpopulation of quiescent cells, glioblastoma initiating cells (GICs), which could be a reason for radioresistance and tumor relapse. The purpose of our study was to investigate if CLIO-ICT has an additive therapeutic effect to enhance the response of GBMs to ionizing radiation.

**Methods:** NSG™ mice bearing human GBMs and C57BL/6J mice bearing murine GBMs received CLIO-ICT, radiation, or combination treatment. The mice underwent pre- and post-treatment magnetic resonance imaging (MRI) scans, bioluminescence imaging (BLI), and histological analysis. Tumor nanoparticle enhancement, tumor flux, microvessel density, GIC, and apoptosis markers were compared between different groups using a one-way ANOVA and two-tailed Mann-Whitney test. Additional NSG™ mice underwent survival analyses with Kaplan-Meier curves and a log rank (Mantel-Cox) test.

**Results:** At 2 weeks post-treatment, BLI and MRI scans revealed significant reduction in tumor size for CLIO-ICT plus radiation treated tumors compared to monotherapy or vehicle-treated tumors. Combining CLIO-ICT with radiation therapy significantly decreased microvessel density, decreased GICs, increased caspase-3 expression, and prolonged the survival of GBM-bearing mice. CLIO-ICT delivery to GBM could be monitored with MRI. and was not significantly different before and after radiation. There was no significant caspase-3 expression in normal brain at therapeutic doses of CLIO-ICT administered.

**Conclusion:** Our data shows additive anti-tumor effects of CLIO-ICT nanoparticles in combination with radiotherapy. The combination therapy proposed here could potentially be a clinically translatable strategy for treating GBMs.

## Introduction

Glioblastoma multiforme (GBM) is one of the deadliest neoplasms in children and young adults, with a median survival of 12-14 months 1, 2. The standard treatment includes maximal safe resection and post-operative chemo/radiotherapy 3-5. When exposed to ionizing radiation, GBM tumor cells undergo DNA damage and tumor cell death 6. However, glioblastoma initiating cells (GICs) pose a challenge to achieving tumor response to radiation therapy. GICs are a subpopulation of quiescent cells that can activate DNA damage repair pathways, escape the toxic effect of radiation and subsequently induce tumor recurrence 7, 8. Increasing doses of radiation have been used to target GICs but systemic toxicity is of significant concern 9. Therefore, finding effective combinations of radiation with other therapeutic methods to target GICs is critical for the development of more effective anti-GBM strategies. The use of nanoparticles, in particular, is attractive given the advantages of improved plasma half-life, bio-availability, and sustained controlled drug release 10.

**Figure 1:**
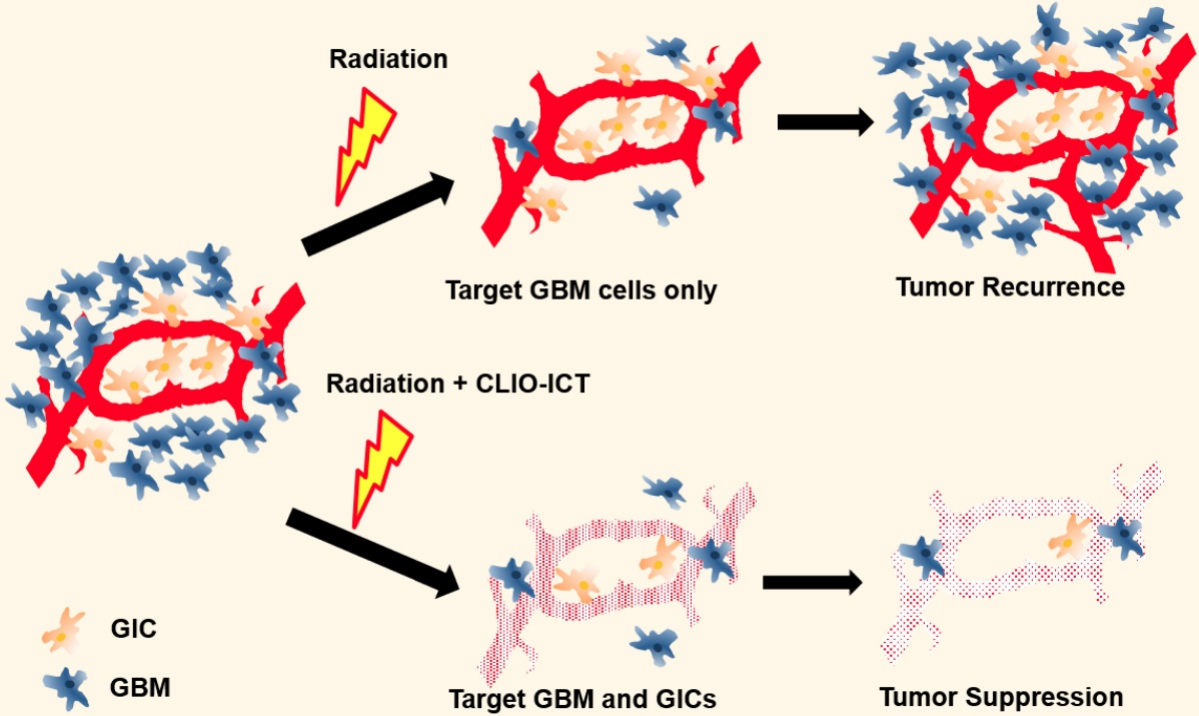
** Illustration of additive effects of CLIO-ICT on radiotherapy.** Radiotherapy targets glioblastoma cells but has a limited effect on glioblastoma initiating cells (GICs). GICs are often radioresistant and important for tumor recurrence. CLIO-ICT targets the vascular niche and indiscriminately induces apoptosis of both highly proliferated GBM cells and quiescent GICs. Therefore, CLIO-ICT could potentially overcome radioresistance and increase the efficacy of radiotherapy in GBMs.

We have recently developed and validated a treatment approach for GBM with theranostic nanoparticles (TNPs) in mouse models (Figure 1) 11. Briefly, the TNPs are composed of FDA-approved cross-linked iron oxide nanoparticles (CLIO) and the highly effective vascular disrupting agent (VDA) azademethylcolchicine (ICT2552). CLIO and ICT2552 are linked through an MMP-14-cleavable linker to afford the MMP-14 activatable theranostic drug, CLIO-ICT 12. Matrix metalloproteinase 14 (MMP-14) is a membrane-bound protein playing an important role in the stemness of aggressive cancers, including GBM 13-15, which makes it an appealing therapeutic target for GICs.

Here, we propose a new strategy of combining theranostic nanoparticles with radiation therapy (RT) for improved treatment of glioblastomas. The theranostic nanoparticles disrupt the blood brain barrier in the tumor tissue through MMP-14 mediated activation of the VDA prodrug. MMP-14 activation releases the vascular disrupting agent, ICT, which initiates a positive feedback loop of nanoparticle uptake and subsequent drug release. BBB breakdown releases the therapeutic drug ICT to the perivascular niche, where GICs are preferentially located. Since MMP-14 is abundant in GBM and not normal brain 13, 16-19, CLIO-ICT provides highly specific destruction of tumor vasculature and highly specific elimination of GBM tumor cells and GICs residing in the perivascular niche. Additionally, the CLIO nanocarrier allows for *in vivo* drug tracking with magnetic resonance imaging (MRI) 11, 12.

Thus, we hypothesize that CLIO-ICT, by targeting GICs, will have additive anti-GBM effects in combination with radiation. In addition, we postulate that CLIO-ICT will improve the efficacy of radiotherapy with marginal toxic effects to the normal brain and visceral organs.

## Methods

**Chemicals and antibodies.** The following antibodies were used: MMP-14 (Santa Cruz), CD31 (Abcam), Desmin (Abcam), CD15 (Abcam), and cleaved caspase-3 (Cell Signaling Technology). The following chemicals were used: Ferumoxytol (AMAG Pharmaceuticals). ICT and TNP were synthesized and characterized according to a previous protocol 11.

**Cell culture.** A patient-derived GBM cell line, GBM39, and a murine GBM cell line, CT-2A, were used *in vitro* and *in vivo*. The cell lines GBM39 and CT-2A were kindly provided by Dr. Sanjiv Sam Gambhir and Dr. Samuel Cheshier (Stanford University). CT-2A cells were grown in DMEM (Life Technologies) containing 10% FBS and 1% Penicillin/Streptomycin (Life Technologies). GBM39 were cultured as floating cellular spheres as previously described 11. All cell lines were authenticated and confirmed to be mycoplasma negative using the MycoAlert Mycoplasma Activity Kit (Lonza). All cell lines used were from early passages and were transfected with firefly luciferase enzyme to enable monitoring of tumor growth using bioluminescence imaging.

**Animal models.** To generate orthotopic GBM xenografts, 300,000 tumor cells were injected stereotactically into the striatum of 8-week-old female NOD scid gamma (NSG™) and C57BL/6J mice, using the following coordinates: 2 mm posterior to the bregma, 2 mm lateral to the midline, and 3 mm deep with respect to the surface of the skull. All animal maintenance, handling, surveillance, and experimentation were performed in accordance with and approval from the Stanford University Administrative Panel on Laboratory Animal Care (Protocol 24965).

**MMP-14 expression in patient tumor and murine tissues.** To confirm that human GBMs have greater MMP-14 expression than normal brain to activate CLIO-ICT, we evaluated the expression of MMP-14 in GBM specimen from 5 chemotherapy-naïve human patients (kindly provided by Dr. Jürgen Schlegel, Neuropathology, Technical University of Munich, Germany) as well as murine GBM specimen from GBM39-inoculated mice using immunohistochemistry (IHC). To estimate CLIO-ICT activation in normal organs, we additionally evaluated MMP-14 expression in murine lung, liver, heart, kidney, and spleen obtained from three C57BL/6J mice. IHC for MMP-14 was performed using the micro-polymer-IHC detection kit (Abcam) following the manufacturer's instructions. Representative images were captured using a Zeiss AxioImager Widefield fluorescence microscope with a 20X objective for whole-slide imaging (evaluated according to Immunoreactivity Score (IRS) system, using the following scores for percentage of positive cells: Negative (0), ≤ 10% (1), ≥ 11% and ≤ 50% (2), ≥ 51% and ≤ 80% (3), ≥ 81% (4).

**Dose response studies.** Fifteen NSG™ mice and fifteen C57BL/6J mice were randomized in five groups (3 mice/group) receiving CLIO-ICT intravenously at different doses (0, 1, 2, 5 and 10 mg/kg ICT; 0, 4.25, 8.5, 17, 34 mg/kg Fe). PBS was administered as vehicle in NSG™ and C57BL/6J mice without CLIO-ICT treatment. Bioluminescence imaging (BLI) was used to determine the anti-tumor effects of CLIO-ICT at various doses. The dose at which we observed maximum inhibition of tumor growth was considered as the optimum therapeutic dose *in vivo.*

**Monitoring tumor growth with BLI.** Tumor formation of the animals were scored by BLI (twice per week after inoculation). Luminescent imaging was performed on an IVIS Spectrum (Caliper Life Science) and quantified using Living Image 4.0 software. D-Luciferin (firefly) potassium salt solution (Biosynth) was prepared (15 mg/mL in PBS) and injected intraperitoneally (90 mg/kg, ~150 µL). Total flux (photons per second) values were obtained by imaging repeatedly until peak radiance was achieved and quantified with Living Image 4.0 software. BLI was repeated at the end of the treatment.

**Toxicity studies.** Toxicity was evaluated by hematoxylin and eosin (H&E) staining, aspartate aminotransferase (AST), alanine aminotransferase (ALT), and creatinine activities. An additional set of thirty non-tumor bearing C57BL/6J mice (6 mice/group) received intravenous injections of PBS, CLIO-ICT (10 mg/kg ICT; 34 mg/kg Fe), CLIO-ICT (70 mg/kg ICT; 238 mg/kg Fe), colchicine (2 mg/kg), or colchicine (10 mg/kg). Given that our theranostic drug is activated in tissue by MMP-14 to release the vasculature disruptive agent, azademethylcolchicine (a colchicine derivative), we used equimolar doses of colchicine, assuming that all CLIO-ICT is cleaved in target tissues. Colchicine 2 mg/kg and 10 mg/kg corresponds to 10 mg/kg ICT and 70 mg/kg ICT, respectively. After day 5 of treatment, blood was collected into heparinized tubes to separate the plasma and stored in -80 ºC. Liver toxicity was assayed using ALT/SGPT Activity Kit (BioVision K752-100) and AST/SGOT Activity Assay Kit (BioVision K753-100) following the manufacturer's instructions. Renal toxicity was assayed using Creatinine (CRE) Assay Kit (MyBioSource MBS2540563) following the manufacturer's instructions. Several organs (liver, heart, kidney, spleen, bone marrow, and brain) were harvested from all animals and fixed in 10% neutral buffered formalin for 24 h, followed by 70% ethanol at room temperature for 24 h. Organs were then embedded in paraffin for 3 h at 67°C. Coronal sections (5 μm thick) were stained with hematoxylin and eosin, and images were acquired with Eclipse E800 camera (Nikon, USA) 11.

**Efficacy of CLIO-ICT or/and radiation combination therapies**. Twenty-eight NSG™ mice and twenty-four C57BL/6J mice with GBM were randomized into five different treatment groups (n = 6 mice/group), which were treated as follows: Group 1: CLIO-ICT treatment (10 mg/kg ICT, 34 mg/kg Fe), Group 2: radiation therapy (10 Gy), Group 3: CLIO-ICT + radiation therapy (same doses as in groups 1 and 2), Group 4: phosphate buffered saline (PBS), and Group 5: temozolomide (TMZ, 33 mg/kg) + radiation therapy. In group 5, two mice died of their brain cancer before completion of the experiment. Once tumor growth had been confirmed by BLI, the above-mentioned therapeutic regimen was administered on day 0 and day 7. Radiation therapy preceded intravenous injection of the therapeutic nanoparticles by 3 hours.

**Computed tomography (CT) and radiotherapy.** Treatment planning and precise delivery of radiation was performed using a PXi X-Rad SmART cabinet irradiator (Precision X-Ray Inc., North Branford, CT). The system has the X-ray tube and the detector plate mounted on U shape gantry that rotates 360° in X and Y plane around the animal stage. The animal stage is supplied by a nose cone delivering isoflurane anesthesia and can move ±10 cm in the X, Y directions and ±15 cm in Z direction. CT images were acquired to facilitate treatment planning using a beam energy of 40 kVp, a beam filter of 2 mm Al and a voxel size of 0.2 or 0.1 mm. Therapeutic irradiations were performed using an X-ray energy of 225 kVp and a current of 13 mA producing a dose rate of 300 mGy/min at the isocenter. A single dose of 10 Gy was delivered using a 10 mm collimator. The treatment planning was performed with an open source RT image software package, version 3.13.1 running on IDL version 8.5.1, where the CT images were used as a reference to decide the beam angle and collimator size 21, 22.

**Magnetic resonance imaging (MRI).** We evaluated tumor delivery of CLIO-ICT in GBM-bearing mice using MRI. GBM39-bearing mice underwent MRI before, 24 hours after, and 14 days after treatment with PBS, radiation, CLIO-ICT, radiation + CLIO-ICT. MRI studies of GBM-bearing mice were performed on a 7T MR scanner (Bruker Biospin, Billerica, MA), using an appropriate surface coil and the following pulse sequences with a field of view of 2 cm × 2 cm and a slice thickness of 0.5 mm: T_2_-weighted fast spin echo (FSE): repetition time (TR): 3476 ms, echo time (TE): 33 ms, flip angle α: 90° and T_2_ multi-slice multi-echo (MSME): TR: 2964 ms, TE: 8, 16, 24, 32, 40, 48, 56, 64, 72, 80, 88 and 96 ms, α: 90°. T_2_ relaxation times of the tumors were calculated as a quantitative measure of tumor contrast enhancement.

**Immunofluorescence.** Mice in groups 1-5 were euthanized after the last imaging procedure for immunofluorescence. Brain tissues were also fixed in 4% paraformaldehyde at 4°C overnight and later immersed in 30% sucrose for 2 days. Afterwards, brains were then embedded in OCT and stored in -80 °C. Coronal sections (5 μm thick) were mounted on superfrost slides, rinsed with PBS and permeabilized with 0.1% Triton X-100 made in PBS solution for 15 min. Subsequently, cells were blocked for 2 h and stained with primary antibodies overnight to determine GIC populations or to evaluate apoptosis upon treatment with radiation and/or CLIO-ICT. The following dilutions were used: CD31 (1:20, Abcam), desmin (1:50, Abcam), CD15 (1:200, Abcam) and cleaved caspase-3 (1:300, Cell Signaling Technology). Nuclei were counterstained with DAPI. Single endothelial cells or clusters of endothelial cells that were positive for CD-31 and desmin were considered as a vessel. Immunofluorescence images were acquired with a Leica SP8 confocal microscope (20X) using Leica AF software. Three “hot spots” from each slide were identified and photographed. The total number and positive number of cells were counted with Image-J in a blindfolded manner and were estimated as a mean ± SD in three different fields from three independent experiments.

**Survival studies.** Mice with GBM39 tumors were treated with CLIO-ICT (10 mg/kg ICT, 34 mg/kg Fe), radiation (10 Gy), CLIO-ICT + radiation, or PBS using the same protocols as above (6 mice/group). Animal survival was evaluated from the first day of treatment until death. Body weight was measured twice a week. To avoid animal suffering, animals were euthanized when meeting predefined criteria, including loss of ability to ambulate, labored respiration, or inability to drink or feed.

**Prussian blue staining.** The biodistribution of CLIO-ICT was investigated by Prussian blue staining. A set of nine mice with GBM39 tumors were treated with PBS, CLIO-ICT (10 mg/kg ICT, 34 mg/kg Fe), or CLIO-ICT (10 mg/kg ICT, 34 mg/kg Fe) + radiation (10 Gy) (3 mice/group). At 72 hours post-treatment, the mice were perfused with PBS and the tumor and visceral organs were harvested. The presence of iron in these specimens was investigated with Prussian blue staining using Iron Stain Kit (Sigma) according to the manufacturer's instructions. Briefly, the tissue slides were deparaffinized and hydrated with deionized water. Subsequently, the slides were placed in working iron stain solution for 10 min. After rinsing with deionized water, the tissue was stained with Nuclear Fast Red solution (Sigma) for 5 min. Lastly, the tissue was rapidly dehydrated with alcohol and xylene and mounted for imaging. Images were acquired with Eclipse E800 camera (Nikon, USA). Three “hot spots” from each slide were identified and were photographed (40X). The blue dots were counted with Image-J in a blindfolded manner and were estimated as a total of number of prussian blue positive cells per 100 cells (%) ± SD in three different fields from three independent experiments.

**Intravital microscopy (IVM).** A calvarial defect with a 5-mm diameter was created in an 8-week-old NOD scid gamma (NSG™) mouse under anesthesia with 2% isoflurane delivered in 100% oxygen. To generate orthotopic GBM xenografts, GBM39 cells (Total = 300,000) were injected stereotactically into the striatum as described above. Prior to implantation, the cells were labeled with CellbriteTM Red (Biotium 30023) according to the manufacturer's instructions. The defect was covered with a cover glass slide and an MR-compatible cranial window chamber to enable real time imaging of tumor cells and tumor cell vasculature. For imaging, the head was immobilized using a stereotactic frame coupled with ear bars (Kopf Instruments, Tujunga, CA). Images were acquired before and after treatment with FITC-labeled CLIO-ICT or CLIO using a microscope (IV-100; Olympus, Tokyo, Japan) with Olympus UplanFL objectives and Olympus FluoView FV300 software. Time per pixel was set to 8-12.5 μsec, and voltage was set to 500-600 V. Images were analyzed with ImageJ software (National Institutes of Health; Bethesda, MD).

**Statistical analysis.** The presence of statistical significance in the data set was determined using one-way ANOVA test. A nonparametric two-tailed Mann-Whitney test was used to compare two groups, unless otherwise specified. Results were presented as mean ± SD. Kaplan-Meier survival curves were compared using the log-rank (Mantel-Cox) test. The level of significance was set at p < 0.05, as compared with the control group. Statistical analyses were carried out with Prism 6.0 software (GraphPad).

## Results

**Development of theranostic nanoparticles.** As previously described, we developed ICT, a peptide-conjugate of the VDA azademethylcolchicine (ICT2552), which is cleaved by MMP-14 to release and activate the therapeutic drug 12, 23. To overcome potential limited tumor retention and systemic toxicity of this small molecular therapeutic drug, we attached the ICT prodrug to the cross-linked iron oxide nanoparticle compound, ferumoxytol 12. CLIO-ICT nanoparticles have a diameter of 21±3 nm (Dynamic Light Scattering) and a zeta potential of 21±7 meV (Laser Doppler Electrophoresis). The number of ICT molecules per iron oxide nanoparticle was determined to be on average 4.7, based on the attached fluorescein absorption in samples with known nanoparticle concentration 12.

**Figure 2:**
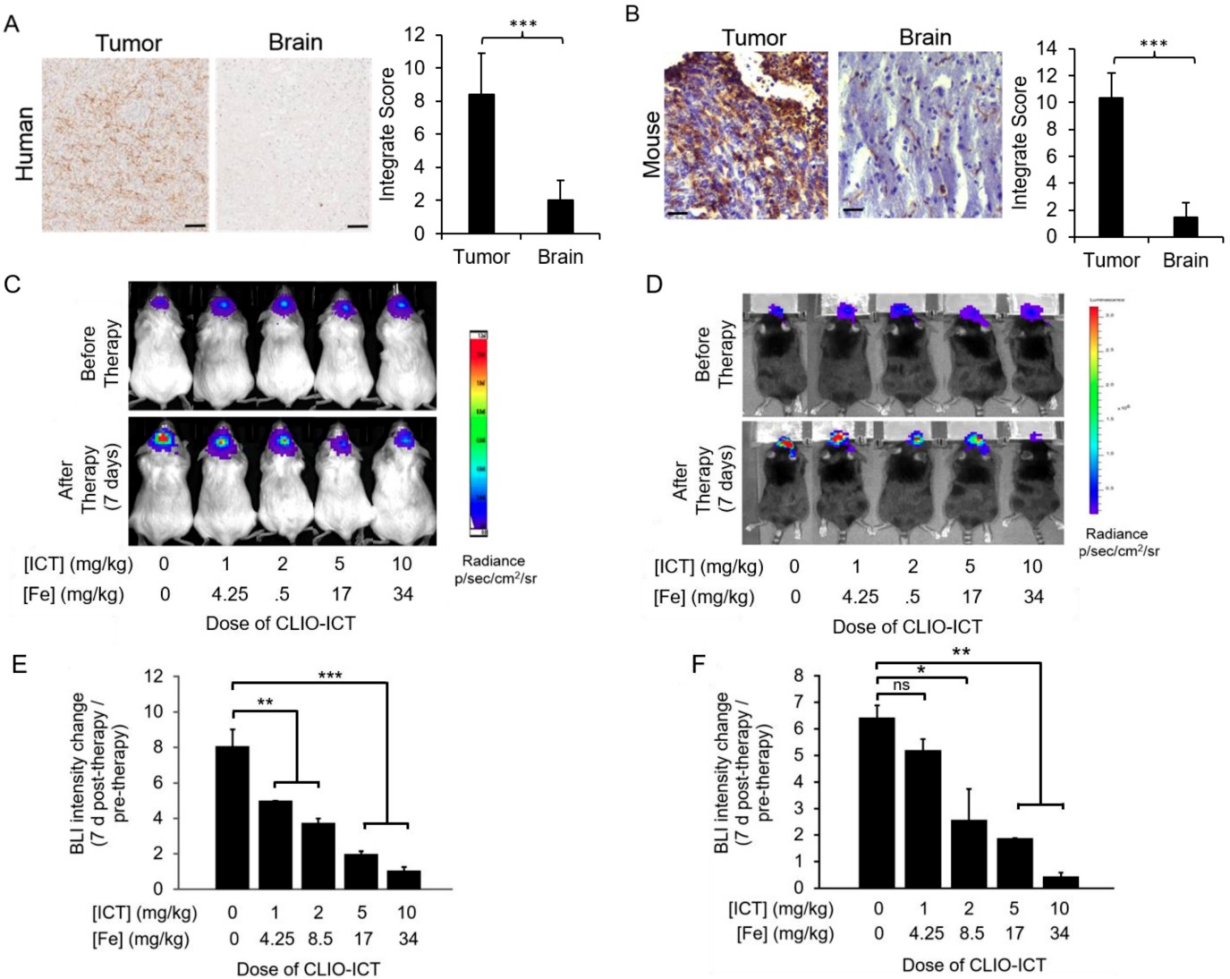
** Dose response studies of CLIO-ICT in human and murine GBMs.** (A) MMP-14 immunohistochemistry staining of human GBM and normal human brain specimen (scale bar, 100 µm). Immunohistochemistry results were evaluated by a semi-quantitative approach. The score for intensity is 0, 1+, 2+, 3+. The percentage of cells is scored : Negative (0), ≤ 10% (1), ≥ 11% and ≤50% (2), ≥ 51% and ≤ 80% (3), ≥ 81% (4). The formula for integrate score: the score for the intensity × the score for percentage of positive cells. (B) MMP-14 staining in murine CT-2A GBM tumors and normal murine brain specimen (scale bar, 40 µm). Immunohistochemistry results were evaluated by a semi-quantitative approach as mentioned above. (C-F) Dose-dependent effects of CLIO-ICT: Bioluminescent *in vivo* images of human GBM39 tumors in NSG™ mice (C) and murine CT-2A tumors in C57BL/6J mice (D). Ratio of bioluminescence radiance before and after therapy for human GBM39 tumors (E) and murine CT-2A tumors (F). Results are represented as the mean ± SD (n = 3 for NSG™ mice, n = 2 for C57BL/6J mice), * p < 0.05, ** p < 0.01, *** p < 0.001, ns = not significant.

**Figure 3:**
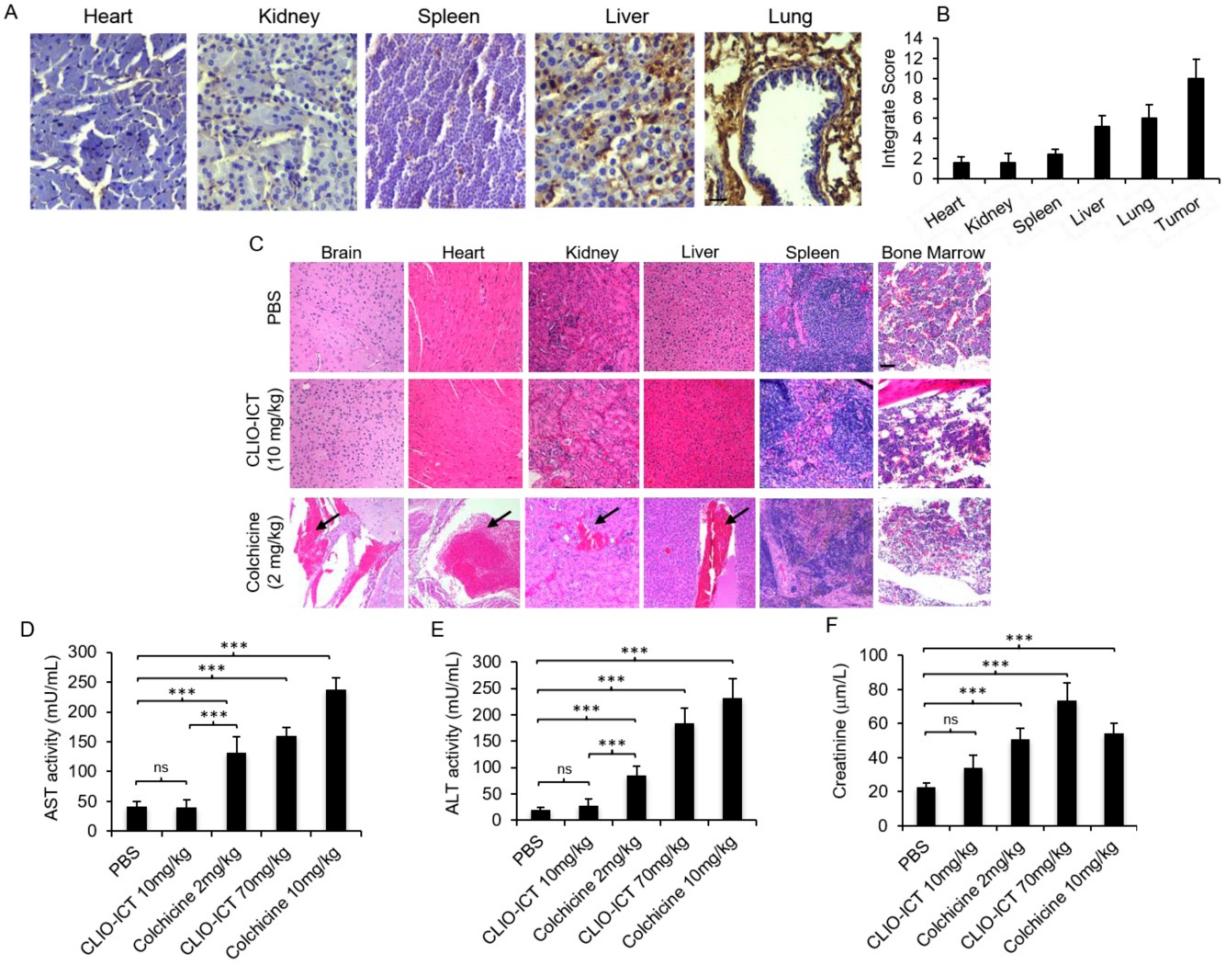
** Safety profile of CLIO-ICT.** (A) MMP-14 immunohistochemistry staining of different organs (heart, kidney, spleen, liver and lung) in C57BL/6J mice (20X magnification). (B) Immunohistochemistry results were evaluated by a semi-quantitative approach as previously described. (C) Representative H&E images show significant necrosis in different organs in colchicine (2 mg/kg) treated mice (n = 6) (10X magnification). Black arrows indicate necrosis. Organs from CLIO-ICT (10 mg/kg ICT) treated mice appear normal and healthy. The graphs show liver toxicity markers, including (D) AST activity, (E) ALT activity, and (F) creatinine concentration in mice treated with PBS, 10 mg/kg or 70 mg/kg CLIO-ICT, and 2 mg/kg or 10 mg/kg colchicine. Results are represented as mean ± SD. * p < 0.05, ** p < 0.01, *** p < 0.001, ns = not significant.

**Expression of MMP-14 in tumor and normal organs.** As a prerequisite for CLIO-ICT activation in GBMs, we first investigated whether human GBMs express MMP-14. IHC confirmed that MMP-14 protein levels in tumor regions of all GBM patient specimens were significantly (p < 0.001) higher compared to normal brain regions (Figure 2A). We confirmed MMP-14 expression in murine GBMs (Figure 2B) and demonstrated CLIO-ICT dose-dependent inhibition of tumor growth in both xenograft and syngeneic GBM models. With a dose of ICT (10 mg/kg) and Fe (34 mg/kg), CLIO-ICT showed the maximum anti-GBM effect in both human (Figure 2C & 2E) and murine GBMs (Figure 2D & 2F) - thus, this optimal dose was used for subsequent combination therapies with radiation. To exclude toxicity in normal organs, we screened tissue specimen of the brain and visceral organs for MMP-14 expression (Figure 3A). MMP-14 expression in the tumor was twice as high as lung and liver and 3-4 times higher than in the kidney, heart, and spleen (Figure 3A & 3B).

**Toxicity studies.** Liver toxicity markers (AST and ALT in plasma) were significantly lower for mice treated with CLIO-ICT compared with mice treated with free colchicine (Figure 3D & Figure 3E, p < 0.0001). Creatinine levels in plasma demonstrated a similar pattern at the therapeutic dose of CLIO-ICT (ICT 10 mg/kg and Fe 34 mg/kg (Figure 3F, p < 0.01). However, excessive doses of CLIO-ICT caused significantly increased creatinine compared to colchicine treated mice (Figure 3D, p < 0.0001). H&E staining further demonstrated signs of necrosis in brain, heart, kidney, liver, spleen, and bone marrow in colchicine treated animals (2 mg/kg, Figure 3C). By comparison, organs from CLIO-ICT-treated animals showed no signs of necrosis (ICT 10 mg/kg and Fe 34 mg/kg, Figure 3C).

**CLIO-ICT biodistribution.** To evaluate CLIO-ICT biodistribution, mice were treated with PBS, CLIO-ICT, or radiation + CLIO-ICT and then perfused with PBS 72 hrs later. Tissues were harvested and stained with Prussian blue to examine the extent of iron uptake in the tumor, brain and visceral organs (Supplementary Figure 1). Compared to PBS treated controls, we found significantly higher iron content in CLIO-ICT treated tumors (p < 0.001). In each treatment group, GBMs demonstrated significantly higher iron uptake than the normal brain regardless of whether the mice were treated with CLIO-ICT alone (p < 0.001) or in combination with radiation (p < 0.001). The degree of iron uptake in the tumor between the monotherapy and combined therapy groups was not significantly different (Supplementary Figure 1) demonstrating consistent delivery of the TNPs to the tumor.

Compared to PBS treated controls, there was no significant iron uptake in the heart, lung, or kidney in mice treated with CLIO-ICT. However, we found significantly higher iron content in the liver of mice treated with CLIO-ICT compared to mice treated with PBS (Supplementary Figure 1). Mice have an intrinsically high iron content in the spleen as one of the spleen's chief roles is to filter blood and recycle iron from aging blood cells. The red pulp of the spleen is also home to mononuclear phagocytes (i.e., macrophages, dendritic cells, and monocytes) which are known to consume iron oxide particles 24. Our results showed that the iron content in the spleen of mice treated CLIO-ICT and PBS was not significantly different. The radiation therapy was collimated to the brain only, therefore, we neither expected nor observed a difference in the iron staining of visceral organs between the CLIO-ICT and CLIO-ICT plus radiation treatment groups.

Preliminary intravital microscopy studies in NSG™ mice with orthotopic GBM3 xenografts showed that intravenously administered CLIO-ICT disrupted the BBB and accumulated in the extravascular space of the tumor tissue (Supplementary Figure 2, green fluorescence). Since MMP-14 is overexpressed on both tumor endothelial cells and tumor cells, CLIO-ICT is activated and released in the tumor vasculature, leading to efficient breakdown of the blood brain barrier (BBB). After intravenous injection of FITC-conjugated CLIO-ICT, the FITC signal in the tumor interstitium was significantly more intense compared to the FITC signal in the tumor interstitium after intravenous injection of CLIO only (without attached vascular disrupting agent). This suggests that CLIO-ICT tumor accumulation is markedly increased due to VDA-mediated vascular disruption. These findings correlated with results of the MRI studies: At 24 h after intravenous injection of CLIO-ICT treatment, tumor T2 relaxation times were significantly shorter compared to CLIO treated controls (p < 0.05). In addition, histology studies at later time points demonstrated tumor vessel disruption and reduced vessel density (quantified as reduced CD31 vessel staining) in CLIO-ICT treated groups compared to CLIO treated groups. CLIO treatment only did not have any effect on tumor vessels, CD31 vessel staining of CLIO treated tumors was not significantly different compared to PBS treated controls (data not shown).

**Figure 4:**
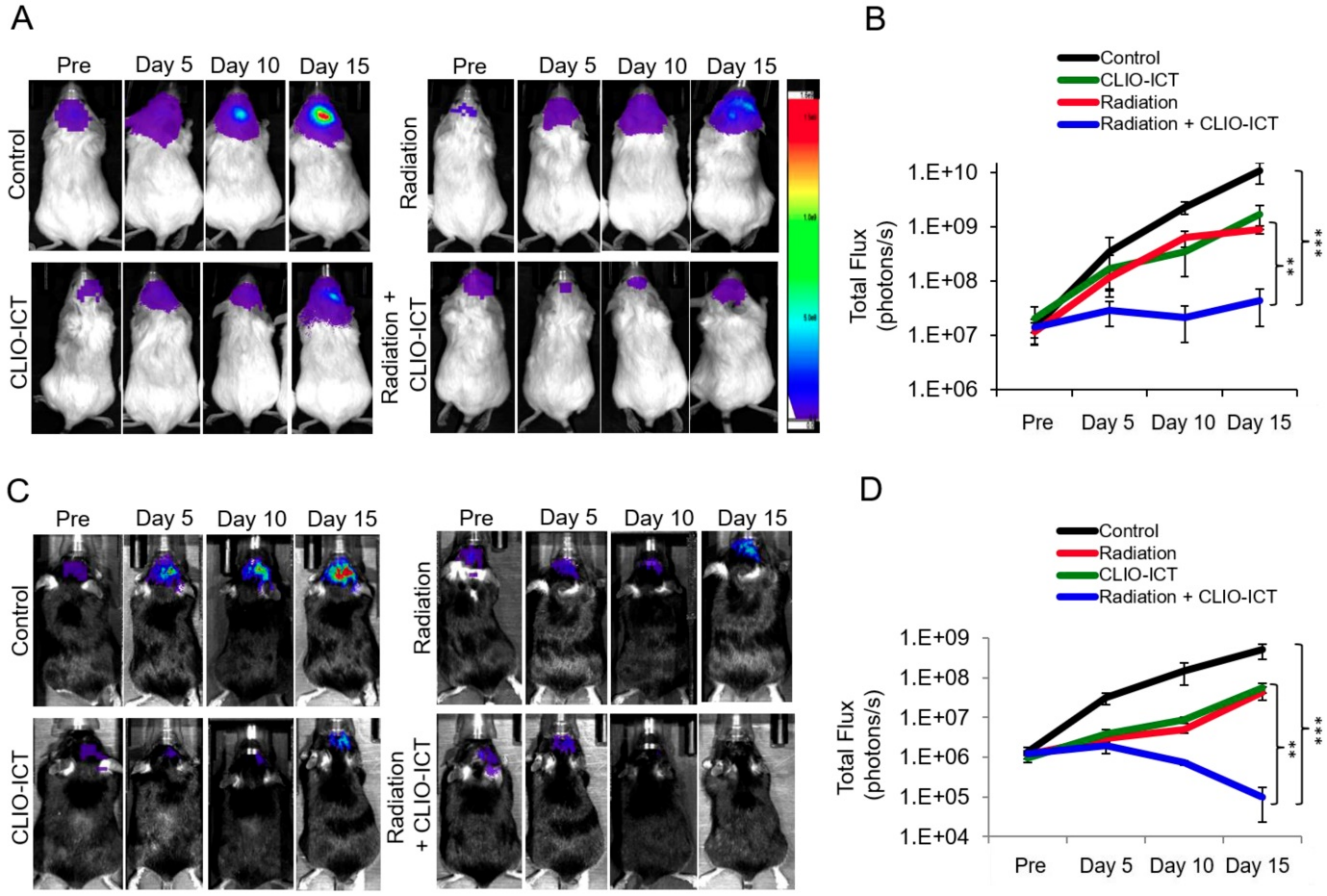
** Efficacy studies of radiation with/or CLIO-ICT therapy *in vivo.***Bioluminescence images of (A) NSG™ mice with orthotopic luciferase-expressing GBM39 tumors or (C) C57BL/6J mice with orthotopic GFP-luciferase-expressing CT-2A tumors. Mice were treated with CLIO-ICT (10 mg/kg ICT, 34 mg/kg Fe) and/or radiation (10 Gy). PBS treatment served as control. Corresponding total flux of tumors before and during treatment are shown for (B) NSG™ and (D) C57BL/6J mice. All mice were treated on Day 0 and again on Day 7. Results are represented as mean ± SD from six animals, * p < 0.05, ** p < 0.01, *** p < 0.001, ns = not significant. Pre = pre-therapy.

**Figure 5:**
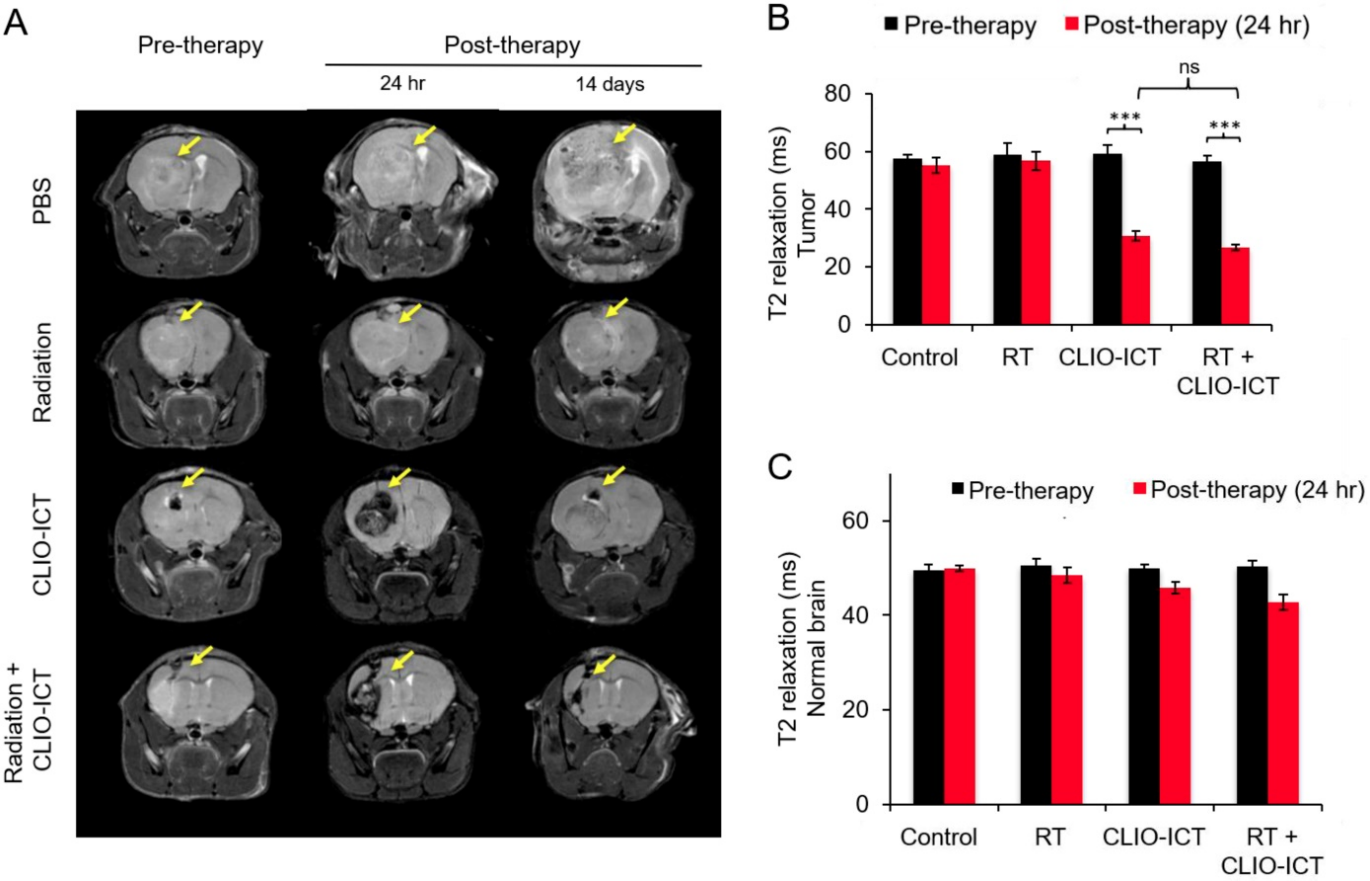
** MR scans after radiation with/or CLIO-ICT therapy.** (A) Representative coronal T2-weighted MR images of the brain of NSG™ mice with GBM39 tumors before and after CLIO-ICT (10 mg/kg ICT, 34 mg/kg Fe) and/or radiation (10 Gy) treatment (24 hr and 14 d). PBS treatment served as control. Theranostic nanoparticle delivery is demonstrated by hypointense (negative/dark) tumor enhancement in CLIO-ICT and CLIO-ICT + radiation treated animals. Yellow arrows indicate the site of tumor growth. (B) Corresponding T2 relaxation times of tumors before and 24 hr after therapy. Note significant shortening of T2 relaxation times of tumors after administration of CLIO-ICT. (C) T2 relaxation times of the normal brain before and 24 hr after therapy. Data represent mean ± SD from six animals, * p < 0.05, ** p < 0.01, *** p < 0.001, ns = not significant.

**Additive effects of CLIO-ICT with radiation therapy.** Next, we assessed whether CLIO-ICT improves the efficacy of radiotherapy for anti-GBM treatment. Two weeks after initiation of treatment, BLI scans revealed significant reduction in tumor size for radiation plus CLIO-ICT-treated GBM39 tumors (Figure 4A & 4B, p < 0.001) and CT-2A tumors (Figure 4C & 4D, p < 0.001) compared to monotherapy or vehicle-treated tumors. Additional mice were treated with the standard of care therapy (TMZ + radiation), which inhibited tumor growth compared to untreated controls. However, the tumor volume of mice treated with TMZ + radiation did not change significantly compared to baseline scans before start of therapy (Supplementary Figure 3). MR scans further demonstrated CLIO-ICT delivery into tumors by a negative (dark) signal enhancement on T2-weighted MR images (Figure 5A & 5B). T2 relaxation times of tumors treated with CLIO-ICT or radiation plus CLIO-ICT were two-fold lower compared to radiation and PBS treated tumors. The T2 relaxation times of GBMs after treatment with CLIO-ICT and CLIO-ICT plus radiation was not significantly different (see Figure 5B, p > 0.05), suggesting that CLIO-ICT delivery to tumors did not change significantly with added radiation. Likewise, T2 relaxation times of normal brain regions were not significantly different between mice treated with CLIO-ICT, radiation plus CLIO-ICT, or PBS suggesting that CLIO-ICT did not accumulate in normal brain before or after radiation therapy (Figure 5C).

**Figure 6:**
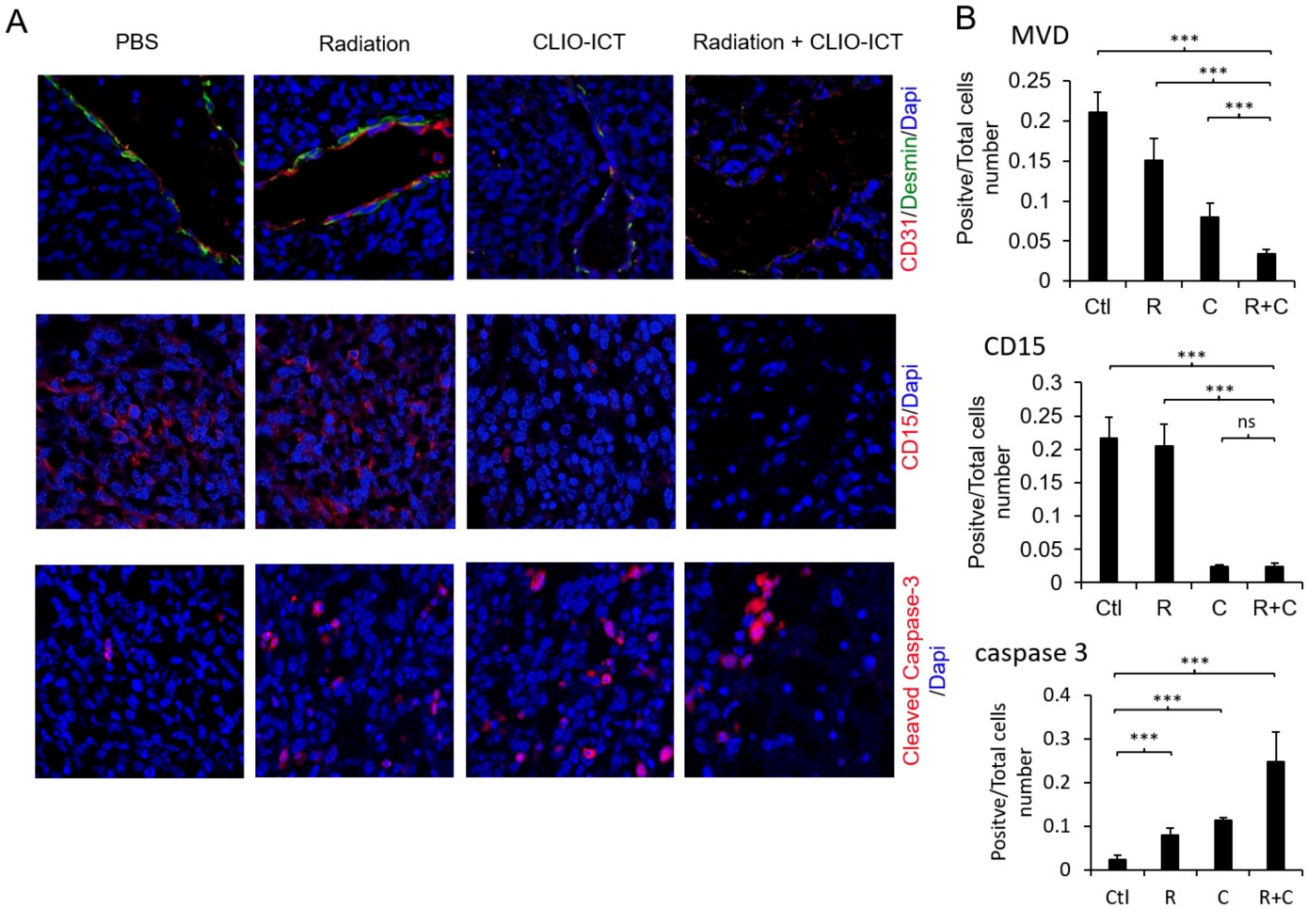
** Effect of mono- or combined-therapy on GBM vasculature and GIC population.** (A) Representative confocal images depicting the effect of mono and combination therapy on tumor vasculature, GICs, and GBM cell death. GBM vasculature is outlined by staining with endothelial cell marker-CD31 and pericyte marker-desmin; GICs are identified using CD15, and GBM cell death is identified with cleaved caspase-3 (20X magnification). (B) Graphs show quantification of tumor microvessel density (MVD), CD15, and cleaved caspase-3 area. Results are represented as mean ± SD from six animals, * p < 0.05, ** p < 0.01, *** p < 0.001, ns = not significant. Tissue sections are from mice that were sacrificed 15 days post-treatment. Ctl = PBS control, R = radiation, C = CLIO-ICT, R+C = radiation + CLIO-ICT.

**Figure 7:**
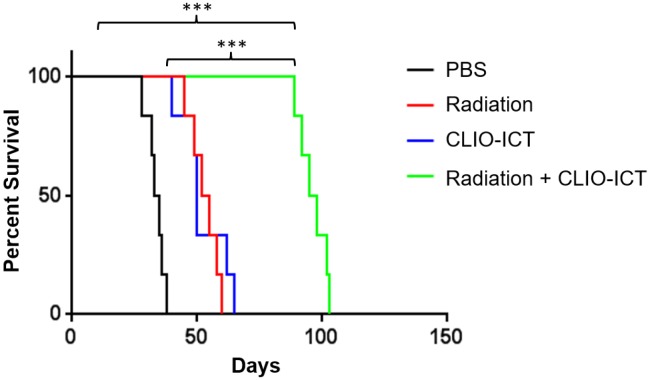
** Radiation and CLIO-ICT combination therapy significantly improves survival of GBM39 tumor-bearing mice.** Kaplan-Meier survival curves demonstrate a significant survival benefit in CLIO-ICT and radiotherapy co-treated group compared with monotherapy or vehicle control (n = 6), log-rank Mantel-Cox test, * p < 0.05, ** p < 0.01, *** p < 0.001, ns = not significant.

Vasculature staining with CD31 and desmin showed reduced vascular density for mice treated with combination therapy (Figure 6A & 6B). The staining for CD15, a known marker for glioblastoma initiating cells 25, also dramatically decreased substantially upon the combination treatment compared to monotherapy and control groups (Figure 6A & 6B). Furthermore, the amount of active caspase-3 increased significantly in radiation and CLIO-ICT co-treated groups compared to the PBS treated control group (p < 0.001), suggesting that apoptosis was induced by the former treatment (Figure 6A & 6B). As expected, we also found that the combination therapy significantly prolonged the survival of GBM-bearing mice compared to control or monotherapy treated animals (Figure 7, p < 0.001). We used H&E staining to confirm that the entire tissue section stained was tumor, which could be recognized by extensive hyperplasia (Supplementary Figure 4).

## Discussion

Despite advances in therapy, the prognosis for GBM patients is still devastating. The recurrence rate of malignant gliomas is approximately 90% 26. Despite aggressive treatment with surgical resection and subsequent chemo/radiation therapy, resistance to adjuvant therapy enhances cancer cell survival, which is a critical cause for tumor relapse 3, 4, 27. While effective for eradicating GBM cancer cells, radiotherapy is less effective for GICs 7, 28. Radiation therapy kills cancer cells by inducing irreparable DNA double-strand breaks, thus highly proliferating cancer cells are more radiosensitive than quiescent GICs. Combining radiation therapy with other cancer therapies has shown benefits over monotherapies, including targeting multiple biochemical pathways, genes, or cell cycle checkpoints to overcome resistance in heterogeneous tumors 29, 30. However, thus far, a curative combination therapy for GBM that can target GICs without added toxic effects has not been found.

Here we investigated a new combined therapeutic approach against GBMs using radiation and the MMP-14-activatable theranostic nanoparticle, CLIO-ICT. We found that the combination therapy demonstrated significant tumor response and prolonged survival time compared to monotherapy or no treatment. The data suggests that CLIO-ICT can improve the therapeutic efficacy of radiation therapy for GBM treatment. There are several examples in the literature where combination therapies involving nanoparticles have enhanced therapeutic outcomes without added toxicity, enhanced efficacy, and overcome drug resistance to elicit sustained treatment responses in cancer patients 30-32. Given our promising results and the fact that CLIO-ICT is based on an FDA-approved iron oxide scaffold, this could potentially be a translatable treatment option for human GBMs.

The combination of CLIO-ICT with ionizing radiation is particularly potent because CLIO-ICT is activated through an MMP-14-cleavable linker and thereby, only activated in the tumor tissue. This limits off-target toxicities to the normal brain. MMP-14 is a membrane-bound protein that plays a role in the degradation of structural proteins in the extracellular matrix and has been identified as an important target for cancer diagnostics and therapy 13, 33. In addition, MMP-14 is also regarded as a marker for GICs 13-15, which makes it a potential target for our drug. As expected, we found a significant reduction in CD15 staining (a marker for glioblastoma initiating cells) in CLIO-ICT and CLIO-ICT plus radiation treated mice, confirming that CLIO-ICT targets cells that have been previously reported as radio-resistant.

One concern with combination therapies is toxicity 34. ICT is a derivative of colchicine, which is a VDA. VDAs are used currently in clinical practice, but they are highly toxic. For example, significant cardiotoxicity has been observed in patients treated with the VDAs DMXAA 35 and combretastatin A4 36, 37. Colchicine is an effective VDA not only because it binds to tubulin and shuts down mitosis but also because it activates platelets which causes serotonin release. Serotonin is also a VDA, acting through G-protein-coupled receptors to generate actin stress fibers and decrease blood flow 38. Our data shows that CLIO-ICT does not accumulate in the heart, because the ICT is covalently linked to nanoparticles which do not extravasate under normal physiological conditions. Our data are in accordance with our previous observations which showed no significant cardiotoxicity after CLIO-ICT treatment 11. While we showed that our CLIO-ICT can potentiate the effect of radiation therapy, radiation therapy can also potentiate the efficacy of our drug. Previous investigators described that, radiation can cause an upregulation of MMPs 39, 40. Greater expression of MMPs would result in enhanced CLIO-ICT activation in the tumor tissue.

VDAs have other advantages to overcome tumor resistance to radiotherapy. It is reported that larger tumors are less radiosensitive than small tumors, which might be due to increased hypoxic regions. VDAs cause a rapid shutdown of perfusion in the established tumor vasculature, leading to tumor cell ischemia and secondary tumor cell death 41-43. These agents have the potential to destroy existing tumor masses and may, therefore, be particularly suitable for treating large tumors 44. Complementarily, conventional cytotoxic radiotherapy has been shown to eradicate a viable rim of tumor cells at the tumor periphery after VDA treatment 45. This observation might be explained by the fact that VDAs are able to kill cancer cells also in hypoxic regions which are less radiosensitive. The remaining rim of viable cells after mono-VDA treatment is believed to be well-oxygenated and thus present an excellent target for conventional cytotoxic radiotherapy. To achieve the strongest additive effect, we treated our animals first with radiotherapy and next with CLIO-ICT (at 3 hours post-radiotherapy), since it has been suggested that blood flow needs to be reestablished to obtain maximum radiosensitization of the tumor 46.

## Conclusion

In summary, intravenous treatment with theranostic CLIO-ICT nanoparticles enhanced the efficacy of ionizing radiation for the treatment of GBM in mouse models in an additive manner, with limited toxicity to the normal brain or visceral organs. In addition, the use of superparamagnetic iron oxide nanoparticles as the backbone of our theranostic drug enabled in vivo drug tracking with MRI. Given the urgent need for new therapies and the shortcomings of current clinical approaches, this new combination therapy provides a new option for GBM treatment. Further studies to enable clinical translation of CLIO-ICT are under way.

## Supplementary Material

Supplementary figures.

## Abbreviations

DMXAA): Glioblastoma multiforme (GBM
