# Supplementary material for: Theranostic nanoparticles enhance the response of glioblastomas to radiation
Source: Nanotheranostics. 2019 Sep 17;3(4):299–310. doi: 10.7150/ntno.35342 (PMC6838141; doi:10.7150/ntno.35342)
Supplement: Supplementary file 1 — Supplementary figures. [file ntnov03p0299s1.pdf]

Supplementary Information

Theranostic nanoparticles enhance the response of glioblastomas to radiation.

Wei Wu[1]†, Jessica L. Klockow[1]†, Suchismita Mohanty[1], Kimberly S. Ku[1], Maryam Aghighi[1], Stavros Melemenidis[2], Zixin Chen[1], Kai Li[1], Goreti Ribeiro Morais[3], Ning Zhao[1], Jürgen Schlegel[4], Edward E. Graves[1,2], Jianghong Rao[1], Paul M. Loadman[3], Robert A. Falconer[3], Sudip Mukherjee[1], Frederick T. Chin[1*], Heike E. Daldrup-Link[1*]

[1] Department of Radiology, Molecular Imaging Program at Stanford (MIPS), Stanford University
[2] Department of Radiation Oncology, Stanford University
[3] Institute of Cancer Therapeutics, Faculty of Life Sciences, University of Bradford, Bradford, UK
[4] Department of Neuropathology, School of Medicine, Technical University of Munich, Munich, Germany
†Co-first author

*__Corresponding authors__:

Heike E. Daldrup-Link, MD, PhD
725 Welch Road, Rm 1665
Stanford, CA 94305-5614
Ph: (650) 723-8996
Fax: (650) 725-8957
H.E.Daldrup-Link@stanford.edu

Frederick T. Chin, PhD
3165 Porter Drive, Rm 2129
Palo Alto, CA 94304
Ph: (650) 725-4182
Fax: (650) 618-0415
chinf@stanford.edu

Table of Contents **Page**

I. **Figure S1**: Prussian blue staining…………………………………………………….....S2

II. **Figure S2:** Intravital microscopy of CLIO and CLIO-ICT-treated mouse…………. S3

III. **Figure S3**: Treatment efficacy of TMZ + radiation…………………………………… S4

IV. **Figure S4**: H&E staining of GBM in mouse brain……………………………………… S5

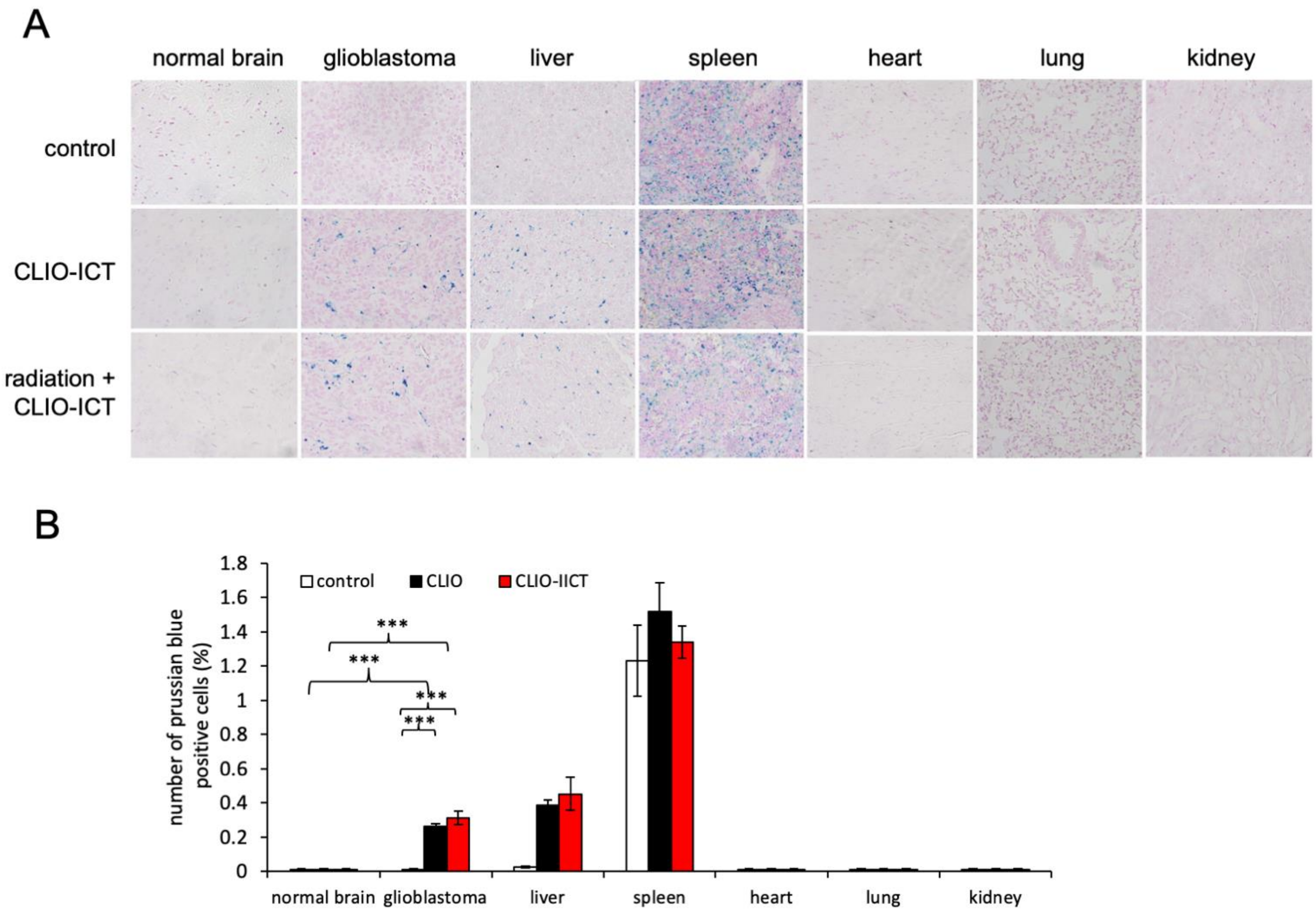

Figure S1: Prussian blue staining. (A) Prussian blue staining of tissues from NSG™ mice in three different treatment cohorts (PBS, CLIO-ICT, or radiation + CLIO-ICT). (B) Quantification of Prussian blue staining showing a significant increase in uptake in the glioblastoma compared to healthy brain. Three "hot spots" from each slide were identified and were photographed (40X). The blue dots and total number of cells were counted with Image-J in a blindfolded manner and were estimated as a total of number of Prussian blue positive cells per 100 cells (%) ± SD in three different fields from three independent experiments.

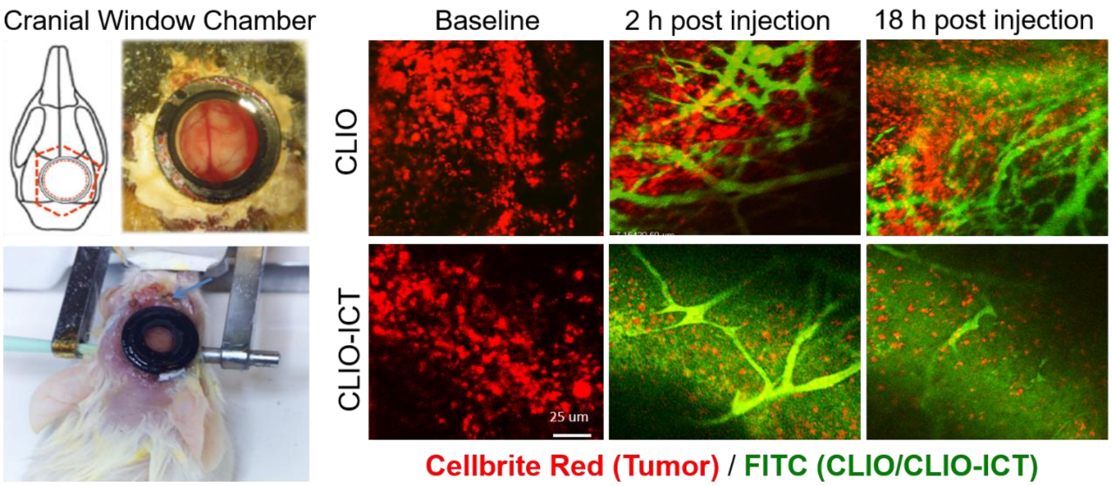

Figure S2: Intravital microscopy of CLIO and CLIO-ICT-treated mouse. An NSG™ mouse bearing a GBM39 tumor was fitted with an intracranial window chamber. The mouse was treated with either CLIO or CLIO-ICT. Time-lapse images were acquired at 2 and 18 h post-injection to study the TNP accumulation and vascular alteration at tumor site. TNPs are labeled with FITC (green fluorescence) and tumors are labeled with Cellbrite Red (red fluorescence).

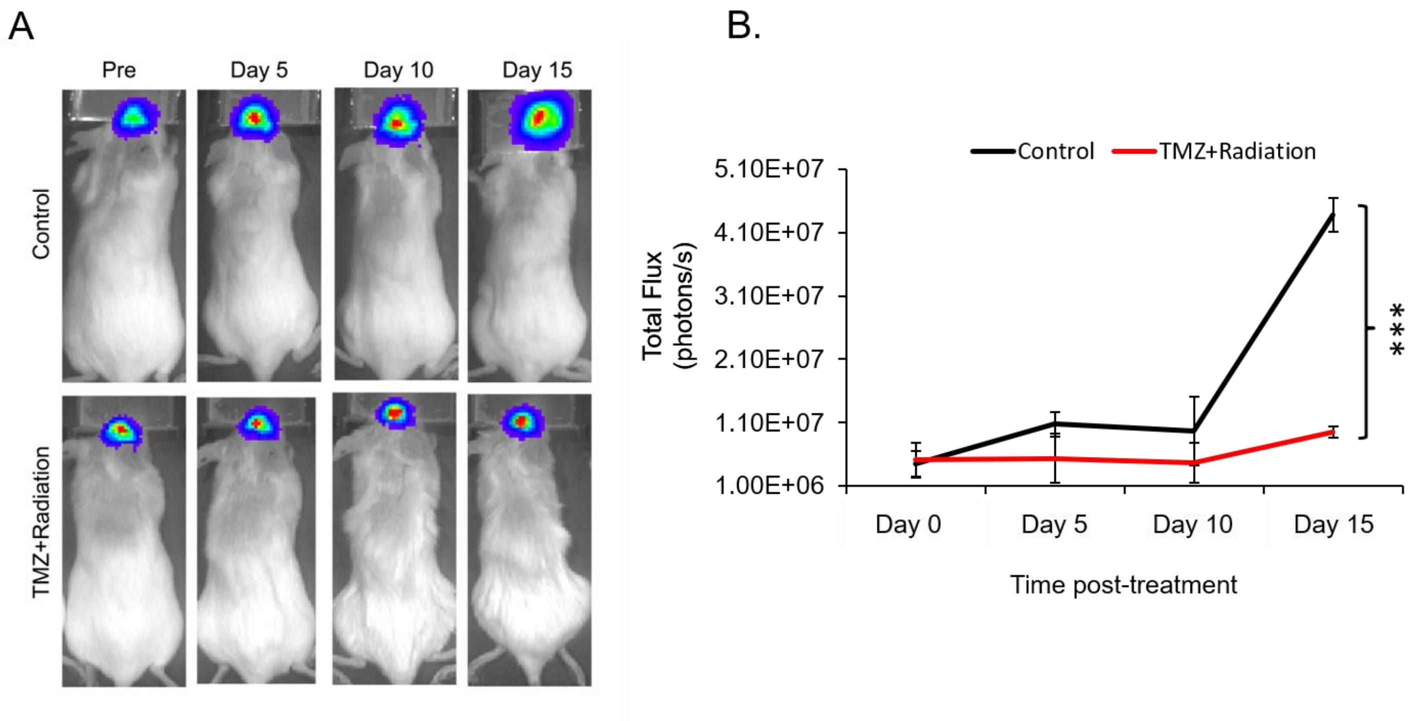

Figure S3. Treatment efficacy of standard of care therapy compared to control. (A) BLI of NSG™ mice that were treated with either PBS or temozolomide (TMZ) and radiation (10 Gy). (B) Graphical representation of tumor size using total photon flux at various time points post-treatment. A significant difference was noted in tumor size between the two treatment groups. (***p < 0.001)

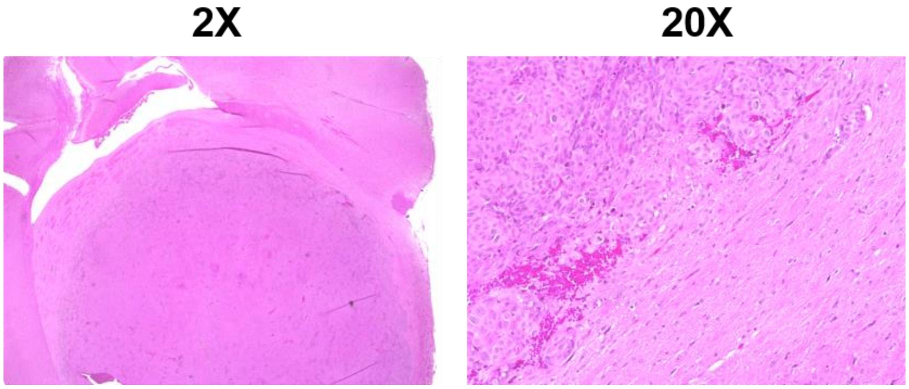

Figure S4. H&E staining of GBM in mouse brain. Staining at two different magnifications shows hyperplasia, a key characteristic of glioblastomas.
